# Supplementary material for: The use of four-pillar regimen for heart failure management: results from the Jordanian Heart Failure Registry (JoHFR)
Source: PeerJ. 2024 Nov 19;12:e18464. doi: 10.7717/peerj.18464 (PMC11583908; doi:10.7717/peerj.18464)
Supplement: Supplemental Information 2 [file peerj-12-18464-s002.docx]

The codes of the SPSS file is in the variables list in SPSS file.
